# Maternal opioid use is associated with altered placental bacterial DNA and activation of immune-apoptotic pathways

**DOI:** 10.1515/nipt-2025-0011

**Published:** 2025-12-16

**Authors:** Yaa F. Abu, Junyi Tao, Arumugam Jayakumar, Hussain Hussain, Michel Paidas, Sabita Roy

**Affiliations:** Department of Surgery, 12235 University of Miami Miller School of Medicine, Miami, FL, USA; Department of Obstetrics, Gynecology and Reproductive Sciences, 12235 University of Miami Miller School of Medicine, Miami, FL, USA

**Keywords:** prenatal opioid exposure, placenta, microbiome, transcriptome, immune activation, maternal-fetal interface

## Abstract

**Objectives:**

Opioid use during pregnancy is associated with adverse perinatal outcomes, but its effects on placental biology are not well understood. Because the placenta plays a vital role in fetal development and immune regulation, we examined how maternal opioid exposure influences microbial DNA signatures and immune gene expression in the placenta.

**Methods:**

Placentas from opioid-exposed and control C57 BL/6 female mice were analyzed through 16S rRNA gene sequencing, bulk RNA sequencing and pathway enrichment analysis.

**Results:**

Opioid-exposed placentas showed altered microbial DNA profiles, including increased α-diversity and enrichment of *Staphylococcus* spp. Transcriptomic analysis revealed 357 differentially expressed genes, emphasizing immune pathways, including dendritic cell-NK cell crosstalk, immunogenic cell death, and cytokine storm signaling. STAT3 signaling and heparan sulfate biosynthesis were downregulated. Pathways related to apoptosis, cytotoxicity, and neonatal death were upregulated.

**Conclusions:**

Maternal opioid exposure may disrupt placental microbial and immune environments, potentially leading to structural compromise through immune-mediated cellular apoptosis.

## Introduction

The presence of bacterial DNA – and in some cases, viable bacteria – in placental and fetal tissues has been reported in numerous human and animal studies [1], [2], [3], [4]. While the idea of a true, resident placental microbiome remains debated, microbial DNA signals detected at the maternal-fetal interface may originate from ascending infection, hematogenous spread, or technical contamination [5], [6], [7]. Regardless of the source, even low levels of microbial DNA in the placenta have been linked to adverse pregnancy outcomes, including fetal growth restriction, preterm birth, and altered neonatal immune development [8], 9]. These findings imply that transient microbial exposure – whether commensal, opportunistic, or pathogenic – may influence placental immune signaling and affect fetal developmental trajectories.

The placenta functions as an immunologically active organ that maintains maternal-fetal tolerance while supporting nutrient transport, gas exchange, and endocrine signaling [10], [11], [12], [13]. Disruptions to this delicate immune balance, especially those caused by environmental stressors, can impair placental function and threaten fetal health [10], [11], [12], [13], [14]. Among these stressors, maternal opioid use during pregnancy has become a major public health concern, with rising rates of *in utero* opioid exposure linked to neonatal abstinence syndrome (NAS) and long-term neurodevelopmental difficulties [15], [16], [17], [18], [19], [20], [21], [22], [23].

Despite increased awareness of these clinical consequences, the molecular mechanisms by which opioids influence placental biology still remain unclear. Opioids are known to have immunosuppressive and proinflammatory effects both peripherally and centrally, including reducing regulatory immune functions and boosting Toll-like receptor signaling [24], [25], [26], [27], [28], [29], [30], [31]. However, whether these effects impact the placenta – such as altering immune tone or microbial recognition – has not been fully elucidated. Additionally, it is unclear to what extent maternal opioid exposure affects bacterial DNA profiles in the placenta and whether these changes are linked to transcriptional dysregulation. To explore this, we conducted an integrative analysis of placental tissue from opioid-exposed and control pregnancies using 16S rRNA gene sequencing, bulk RNA sequencing, and pathway enrichment analysis to better understand how maternal opioid exposure might disrupt placental function and contribute to adverse developmental outcomes.

## Methods

### Animals

All procedures involving animals were performed in accordance with ethical guidelines for laboratory animal care and received approval from the Institutional Animal Care and Use Committee (IACUC) at the University of Miami. Twelve-week-old female C57BL/6 wild-type (WT) mice (20–24 g) were obtained from Jackson Laboratories (Bar Harbor, ME, USA; strain #003752). Age-matched littermate WT mice served as controls. The animals were housed in groups of five in sterile microisolator cages under specific pathogen-free conditions, with controlled temperature (22±2 °C), humidity (30–70 %), and a 12-h light/dark cycle (lights on at 07:00). Standard chow and water were provided *ad libitum*. All procedures were designed to minimize animal discomfort and to limit the number of animals used. Euthanasia was performed using CO_2_ asphyxiation followed by cervical dislocation, in accordance with the American Veterinary Medical Association (AVMA) Panel on Euthanasia guidelines. Experiments complied with the ARRIVE guidelines, the U.K. Animals (Scientific Procedures) Act of 1986, and the National Research Council’s Guide for the Care and Use of Laboratory Animals.

### Experimental design

Twelve-week-old female C57BL/6 mice were bred with age-matched male C57BL/6 mice at a ratio of two co-housed females per one male per cage. After five days of co-housing, males were removed. Females were monitored daily for copulation plugs to confirm mating and accurately determine the start of pregnancy. The day a copulation plug was observed was designated as embryonic day (E) 1. Females that did not exhibit a plug or become pregnant within the 5-day mating period were excluded from the study. Pregnant mice were then randomly assigned to receive subcutaneous injections of either saline or morphine (15 mg/kg, s.c., twice daily) from embryonic days 14–18. On embryonic day 18, dams were humanely euthanized, and placental tissue and ileal contents were collected for downstream fecal and transcriptomic analysis. Placentas were collected from five dams per experimental group, with five placentas harvested from each dam (n=25 placentas per group).

### 16S rRNA gene sequencing

DNA was extracted from fecal samples under aseptic conditions using the DNeasy 96 PowerSoil Pro QIAcube Kit with the QIAcube HT liquid-handling system (Qiagen, Maryland, USA). Two extraction controls were included to check for contamination during processing. Sequencing was performed at the University of Minnesota Genomics Center. The hypervariable V4 region of the 16S rRNA gene was PCR-amplified using the forward primer 515F (GTGCCAFCMGCCGCGGTAA) and reverse primer 806R (GGACTACHVGGGTWTCTAAT), along with Illumina adaptors and molecular barcodes, to generate 427 base pair (bp) amplicons. These amplicons were sequenced on the Illumina MiSeq v3 platform, generating 300-bp paired-end reads. The extraction controls failed to amplify by PCR and were therefore excluded from the sequencing process.

### Microbiome analysis

Demultiplexed sequence reads were processed and clustered into amplicon sequence variants (ASVs) using the DADA2 package (version 1.26.0) [32] implemented in R (version 4.2.3) and RStudio (Build 353). The DADA2 pipeline includes error filtering, trimming, error rate learning, denoising, merging of paired-end reads, and chimera removal. During trimming, forward and reverse reads were truncated at positions 220 and 180, respectively, to remove low-quality base pairs. Taxonomic classification of ASVs was performed to the species level using DADA2’s naive Bayesian classifier [33] with the Greengenes reference database (version 13.8) [34]. ASV and taxonomy tables are provided in Table S1. These tables were imported into MicrobiomeAnalyst [35] for downstream analysis, including alpha and beta diversity plots, taxonomic bar plots, and Linear Discriminant Analysis Effect Size (LEfSe) plots [36]. ASVs with low counts and low variance were filtered using default thresholds. Total sum scaling was applied for normalization. The logarithmic LDA score threshold for discriminative features was set to 2.0. Multiple testing correction was performed using the Benjamini–Hochberg method, with a q-value cutoff of 0.1 for LEfSe analysis. Statistical analysis was conducted using the Mann–Whitney U test to assess differences in α-diversity between treatment groups. Differences in β-diversity were assessed using permutational multivariate analysis of variance (PERMANOVA).

### RNA quantification and sequencing

Ileal tissue was collected from pregnant animals after sacrifice, immediately flash-frozen in liquid nitrogen, and stored at −80 °C until processing. Total RNA was extracted using TRIzol reagent (Invitrogen, Carlsbad, CA, USA) following the manufacturer’s instructions. RNA quality was assessed using: 1 % agarose gel electrophoresis to evaluate degradation and contamination, spectrophotometric analysis with the NanoPhotometer (IMPLEN, Westlake Village, CA, USA) to determine RNA purity and concentration, and fluorometric quantification with the Qubit RNA Assay Kit (Life Technologies, CA, USA). RNA integrity was further evaluated using the RNA Nano 6000 Assay Kit on the Agilent Bioanalyzer 2100 system (Agilent Technologies, CA, USA). Samples passing quality control underwent poly(A) enrichment and library preparation using the NEBNext^®^ Ultra™ RNA Library Prep Kit for Illumina^®^ (New England Biolabs, USA), following the manufacturer’s protocol. Paired-end sequencing (2 × 150 bp) was carried out on the Illumina HiSeq 4000 platform by Novogene Bioinformatics Technology Co., Ltd. (Beijing, China). Sequencing reads were aligned to the mouse genome assembly GRCm39.

### Transcriptome analysis

All raw RNA-seq reads were processed by Novogene Co., Ltd. Raw FASTQ files were first quality-filtered using *fastp* (v0.22.0) [37], which removed adapter sequences, reads containing poly-N, and low-quality reads to generate clean data. The reference genome and gene annotation files (GRCm39) were obtained directly from Ensembl (http://useast.ensembl.org/index.html). Genome indexing was performed using *HISAT2* (v2.2.1) [38], and clean paired-end reads were aligned to the reference genome with *HISAT2*. Read counts per gene were quantified using the *featureCounts* function [39] from the *Subread* package (v2.0.6). Differential gene expression analysis between experimental groups was conducted using the *DESeq2* package (v1.40.2) in R [40]. p-values were adjusted using the Benjamini–Hochberg method to control for false discovery rate (FDR), and genes with adjusted p-values ≤0.05 were considered differentially expressed. Canonical and disease pathway analysis was conducted using Ingenuity Pathway Analysis (IPA, Qiagen; https://digitalinsights.qiagen.com/products-overview/discovery-insights-portfolio/analysis-and-visualization/qiagen-ipa/) on genes with FDR<0.05. Gene Set Enrichment Analysis (GSEA) of Kyoto Encyclopedia of Genes and Genomes (KEGG) [41] pathways was performed using the *clusterProfiler* R package (v4.8.2) [42], with KEGG terms considered significantly enriched at adjusted p<0.05. Visualization of the morphine and codeine metabolism pathway was generated using the *Pathview* R package (v1.42.0) [43].

### Materials

Morphine was obtained from the National Institutes of Health [NIH]/National Institute on Drug Abuse [NIDA] (Bethesda, MD).

## Results

### Bacterial DNA is detectable in placental tissue following opioid exposure

**Figure 1:**
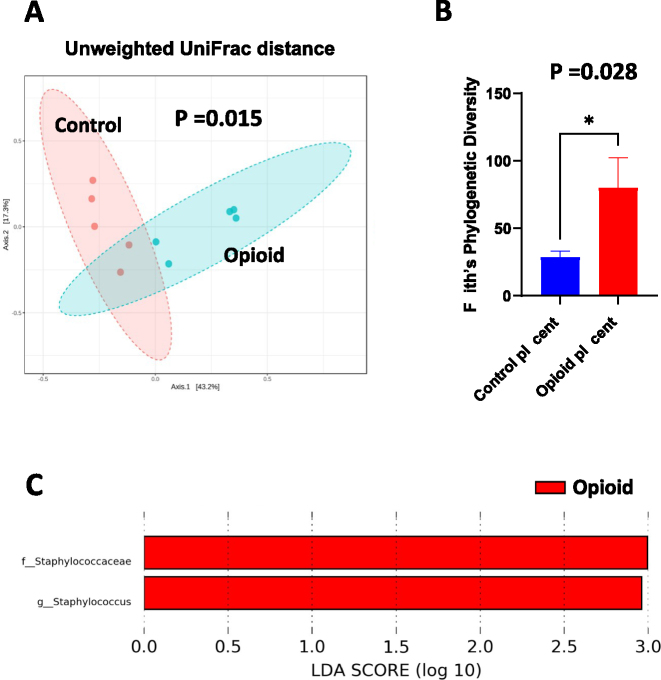
Detection of bacterial DNA in opioid-exposed placental tissue, (A) principal coordinates analysis (PCoA) plot of unweighted UniFrac distances (β-diversity metric). p-value = 0.015. (B) Faith’s phylogenetic diversity (α-diversity metric). Error bars indicate SEM. (C) LEfSe (Linear Discriminant Analysis Effect Size) analysis of the top discriminative bacterial taxa between opioid-treated and control placentas. Samples were grouped as control (n = 5) and opioid (n = 5).

To determine whether maternal opioid exposure is linked to variations in bacterial DNA signatures in the placenta, 16S rRNA gene sequencing was conducted on placental tissues from both opioid-exposed and control murine pregnancies. Analysis of bacterial profiles showed notable differences in community composition and diversity between the groups. β-diversity analysis using unweighted UniFrac distances, visualized through principal coordinate analysis (PCoA), showed distinct clustering of samples from opioid-exposed versus control mice (PERMANOVA, p<0.01; Figure 1A), indicating compositional shifts. Furthermore, α-diversity, measured by Faith’s Phylogenetic Diversity, was significantly higher in placentas from opioid-exposed pregnancies compared to controls (p<0.05; Figure 1B), implying increased phylogenetic richness. Linear Discriminant Analysis Effect Size (LEfSe) identified specific bacterial taxa enriched in the opioid-exposed group. Notably, the family *Staphylococcaceae* and genus *Staphylococcus* showed significantly higher relative abundance in placentas from opioid-exposed mice (Figure 1C). No taxa were found to be differentially enriched in control samples. These findings suggest that maternal opioid exposure may affect the presence, abundance, or translocation of bacterial DNA in the placenta.

### Opioid exposure induces immune-related transcriptomic changes in the placenta

**Figure 2:**
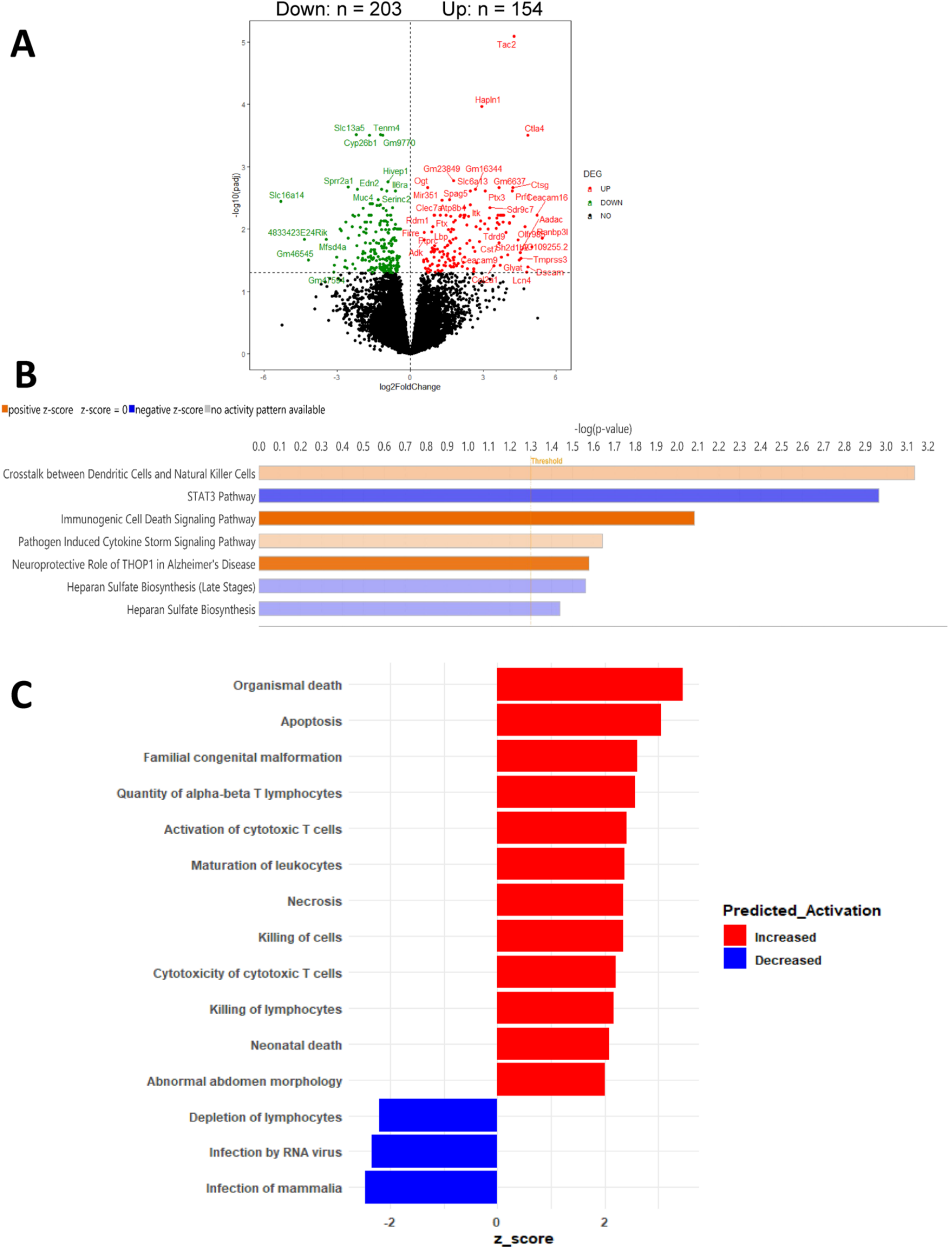
Opioid exposure induces immune-related transcriptomic changes in the placenta, (A) volcano plot of 357 differentially expressed genes between opioid and control group (Padj<0.05). (B) Barplot of significant ingenuity canonical pathways between opioid and control group (p<0.05). (C) Barplot of significant disease pathway between opioid and control group (p<0.05).

Although the placenta is not regarded as a stable site for microbial colonization, bacterial DNA and other low-biomass microbial signatures have been detected in both healthy and diseased pregnancies, where they are believed to influence local immune activity. To assess the overall transcriptional effect of opioid exposure on placental tissue, bulk RNA sequencing was conducted on samples from opioid-exposed and control pregnancies. A total of 357 differentially expressed genes (DEGs) were identified, including 203 genes that were upregulated and 154 that were downregulated in the opioid group (adjusted p<0.05, |log_2_FC|>1; Figure 2A; Table S2). Principal component analysis showed that gene expression profiles clustered according to exposure condition, indicating that opioid exposure produces a consistent and distinguishable transcriptional signature in the placenta.

To determine the biological relevance of the observed DEGs associated with gestational opioid exposure, pathway enrichment analyses were conducted using Ingenuity Pathway Analysis (IPA) and Gene Set Enrichment Analysis (GSEA). IPA identified 24 canonical pathways that were significantly affected by opioid exposure (p<0.05; Figure 2B; Table S3). The most strongly upregulated pathways were Crosstalk Between Dendritic Cells and Natural Killer Cells, Immunogenic Cell Death Signaling Pathway, and Pathogen-Induced Cytokine Storm Signaling. Specifically in the Crosstalk Between Dendritic Cells and Natural Killer Cells pathway, PRF1 (perforin 1) and FASLG (Fas ligand) were among the most upregulated genes, with an 18- and 6.5-fold increase, respectively (Figure S1). Both perforin and Fas ligand play an important role in inducing apoptosis in the target cell. In immunogenic cell death signaling pathway, *PRF*1 and multiple granzyme genes were most upregulated, with fold increases from 14 to 64 (Figure S3). Granzymes activate the caspase cascade within the target cell, leading to the cell’s fragmentation and ultimate apoptosis. In pathogen-induced cytokine storm signaling, PRF1, FASLG and EOMES (eomesodermin) were the top upregulated genes (Figure S4). The upregulated genes and pathways suggested a convergence of inflammatory, innate immune, and neuroimmune signaling. Conversely, suppression was noted in immunomodulatory and structural maintenance pathways, such as the *STAT3 Pathway* and *Heparan Sulfate Biosynthesis*. In STAT3 pathways, SOCS1 (suppressor of cytokine signaling), CSF2RB (colony stimulating factor 2 receptor) and IL22RA1 (interleukin 22 receptor subunit alpha) were the most downregulated genes (Figure S2). Downregulation of those cytokine receptors and growth factor receptors led to a decrease in STAT3, which in turn decreased protection against apoptosis. The downregulated genes and pathways are consistent with the upregulated findings, both reflecting an increase in immunogenic induced cell death and apoptosis.

Additionally, a total of 15 downstream disease pathways related to immune activation and disruption of developmental programs were predicted to be differentially expressed (Figure 2C; Table S4). Pathways related to immune function were activated, including *Apoptosis*, *Organismal Death*, *Necrosis*, *Neonatal Death*, *Cytotoxicity of Cytotoxic T Lymphocytes*, *Maturation of Leukocytes*, and *Killing of Lymphocytes*, as well as cellular and anatomical processes such as *Abnormal Abdomen Morphology*. These changes may indicate that immune cytotoxicity and cell death programs are broadly engaged in the opioid-exposed placenta. Conversely, pathway related to *Depletion of Lymphocytes* was decreased, potentially reflecting either a shift in immune cell function or suppression of anti-pathogen responsiveness.

**Figure 3:**
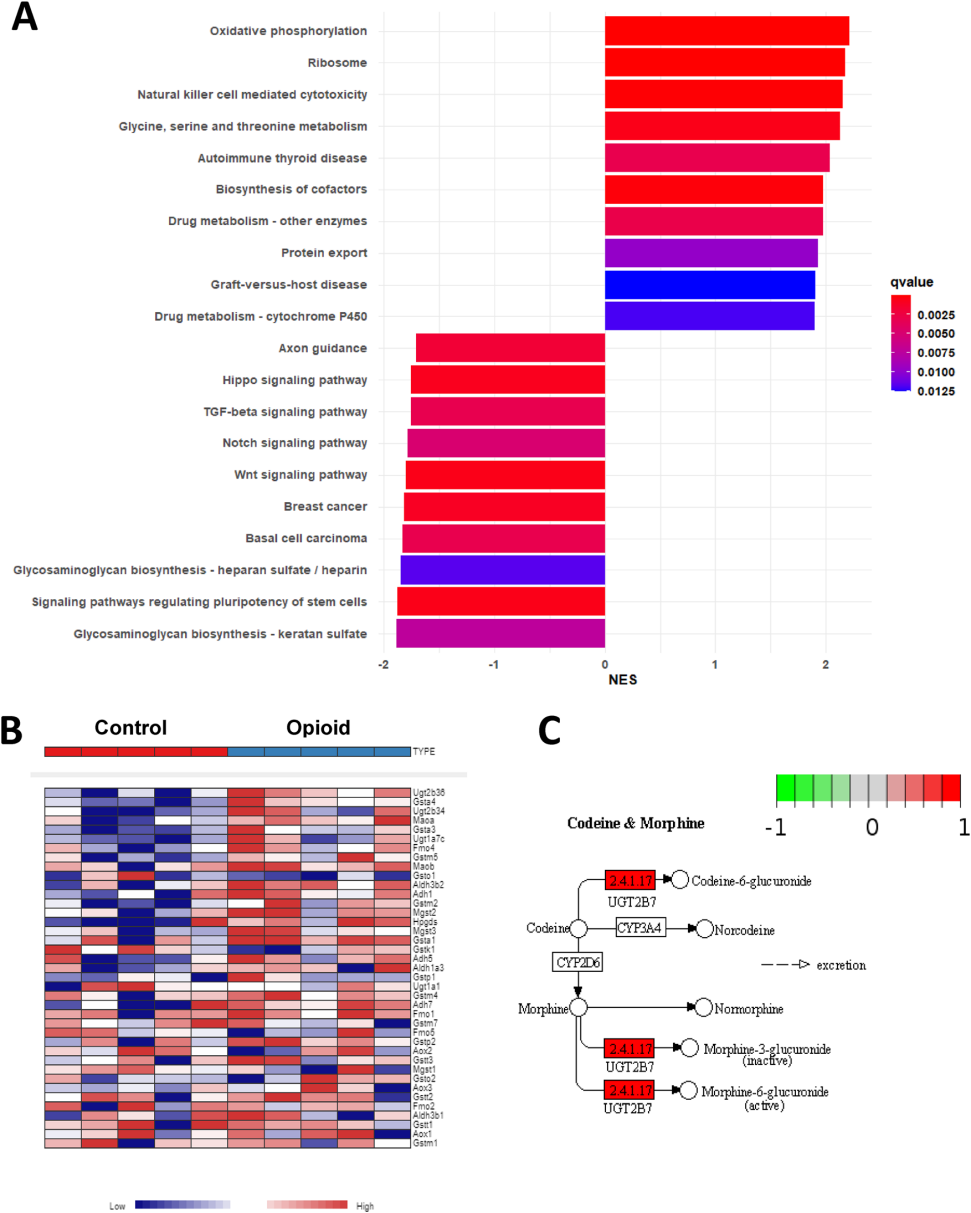
Enrichment of immune pathways and suppression of regulatory signaling in opioid-exposed placentas, (A) Gene Set Enrichment Analysis (GSEA) of KEGG pathways enriched in opioid and control groups. The top 10 pathways (ranked by NES) are shown. (B) Heatmap of genes in the drug metabolism – cytochrome pathway from the KEGG database. (C) Pathway map of morphine and codeine metabolism generated from pathview software.

Complementary GSEA using KEGG annotations identified 55 significantly enriched pathways (adjusted p<0.05; Figure 3A; Table S5). The top 10 increased and decreased pathways ranked by normalized enrichment score (NES) were plotted in Figure 3A. Immune pathways such as *Natural Killer Cell-Mediated Cytotoxicity* were enriched in opioid-exposed samples, consistent with the IPA results. In addition, pathways related to xenobiotic metabolism, including *Drug Metabolism – Cytochrome P450*, were also activated. Within this pathway, expression of **UGT2B7**, a key enzyme in morphine and codeine metabolism, was significantly increased (Figure 3B and C). Taken together, these findings demonstrate that opioid exposure during pregnancy induces significant alterations in placental expression of key genes related to drug metabolism, immune modulation, neurodevelopment, and organ growth, which may be linked to functional impairments later in life.

## Discussion

Opioid use is increasing among women of reproductive age [23], 44]. Opioids readily cross the placenta, leading to fetal exposure and potential long-term effects on immune development, neurodevelopment, and growth [22], 45], 46]. Given the placenta’s central role in maternal-fetal exchange and immune regulation, disruptions in its function may underlie some of these adverse outcomes [47]. In this study, we examined how maternal opioid exposure alters placental biology. Building on prior evidence that opioids induce microbial dysbiosis and modulate immune responses, we examined whether opioid exposure is associated with the presence of bacterial DNA in the placenta and whether these changes are linked to transcriptomic alterations in placental tissue.

Our previous work, along with others, has linked opioid use during pregnancy to microbial dysbiosis in the maternal gut, breast milk, and infant gut [48], [49], [50], [51]. In this study, we additionally detected bacterial DNA – predominantly *Staphylococcus* – in placentas from opioid-exposed animals. As 16S rRNA sequencing cannot differentiate between viable and non-viable bacteria or confirm active colonization, the observed profiles likely reflect translocation events or changes in immune barrier function, rather than stable microbial communities. However, contamination cannot be completely ruled out, especially given the low biomass of placental tissue. To our knowledge, no earlier studies have examined bacterial DNA in opioid-exposed placentas, underscoring the need for more research with proper controls and validation methods.

We further assessed how opioid exposure might reshape the placental transcriptome in the context of bacterial DNA presence. RNA-seq analysis revealed enrichment of several immune-related pathways, including dendritic cell-NK cell signaling, pathogen induced-inflammatory cytokine responses, and regulated cell death. Perforin, Fas ligand, and granzymes were the key genes that drived the enrichment of those pathways. All of them are the key molecules utilized by NK cells to induce apoptosis in target cells [52], 53]. These changes suggest a state of heightened immune activation, potentially driven by increased microbial translocation (as shown in our 16S rRNA sequencing result) or direct opioid-induced immune dysregulation. It is important to emphasize that the present study cannot determine whether the bacterial DNA signatures contributed to the transcriptional changes or whether both findings independently result from opioid exposure. The associations observed here are correlative, and the study was designed to examine the independent effects of *in utero* opioid exposure on placental bacterial DNA and immune profiles rather than to establish a causal relationship between microbial DNA and gene expression. As an exploratory study, these findings should be interpreted as hypothesis-generating and will require mechanistic follow-up analyses. We also observed upregulation of neuroimmune signaling pathways, which may link placental inflammation to downstream neurodevelopmental risks in exposed offspring. Conversely, key regulatory and structural pathways were suppressed, including those involving STAT3 signaling and extracellular matrix biosynthesis. STAT3 is essential for maintaining immune balance, inhibiting apoptosis [54] and supporting vascular development [55], while heparan sulfate modulates growth factor signaling and contributes to extracellular matrix integrity [56]. The downregulation of these pathways suggests a disruption of placental homeostasis, consistent with a pro-inflammatory, pro-apoptosis and structurally compromised environment.

Our findings align with previous studies of human placentas exposed to opioids [57], [58], [59]. For example, Sousa et al. [59] reported many enriched pathways that were also highlighted in our analysis [59]. They identified Natural Killer Cell-mediated Cytotoxicity as one of the top enriched pathways using KEGG database, which is consistent with the results from our KEGG GSEA analysis (Figure 3A) and IPA pathway analysis (Figures 2B and S1, S3). In addition, pathways related Drug Metabolism – Cytochrome P450 were activated in their study and similarly observed in our analysis (Figure 3A). Moreover, their finding of genes involved in apoptosis coincided with our analysis (Figures 2B and S3). It is important to note that some pathways identified by automated tools such as IPA and KEGG, particularly those traditionally associated with neuronal processes (e.g., axonal guidance) are driven by genes that have broad cellular functions and are not specific to neural tissue. While these pathways appear in the analysis due to shared signaling molecules, they should not be interpreted as evidence of neuronal development occurring within the placenta. Accordingly, we have limited our interpretation to pathways with clear relevance to placental immune activity, apoptosis, structural integrity, and general cellular function. A key advantage of our preclinical model is the ability to isolate opioid-specific effects on the placental transcriptome. Unlike human studies, which often involve confounding exposures to non-prescribed substances or co-medications, our approach eliminates these variables, providing clearer insight into the direct impact of opioids on placental biology. This advantage likely enabled us to identify a greater number of DEGs using a more robust statistical threshold (adjusted p-value <0.05) compared to the threshold employed by Sousa et al. (adjusted p-value <0.25). However, a limitation of this study is that placentas were not separated by fetal sex, preventing analysis of sex-specific transcriptional responses. Future investigations should address this gap, as sex-based differences may further shape placental responses to opioid exposure. Additionally, in this exploratory study, five placentas were collected from each dam, which limits biological independence because placentas from the same pregnancy are not fully independent replicates. Future experiments will increase the number of dams per group so that no more than two placentas per dam are used for downstream analyses, thereby improving rigor and statistical independence.

Overall, these results support the conclusion that opioid exposure alters placental immune signaling and development. Our findings align with prior research indicating that prenatal opioid exposure is linked to adverse pregnancy outcomes like fetal growth restriction and preterm birth – conditions often associated with placental insufficiency [60], [61], [62]. Although the underlying mechanisms are not fully understood, early histological reports have described increased fibrin deposition, irregular vascular remodeling, and excessive trophoblast proliferation in opioid-exposed placentas – findings indicative of chronic hypoxia and impaired perfusion [63]. One possible mechanism involves the dysregulation of placental opioid receptors; chronic opioid exposure may desensitize or downregulate these receptors, which are believed to modulate placental immune responses, hormone signaling, and vascular tone [64], [65], [66]. While further research is necessary to clarify the specific roles of these receptors during pregnancy, their disruption could contribute to the transcriptomic abnormalities observed in this study. Although our study focused on late-gestational opioid exposure to isolate opioid-specific effects on placental immune and microbial signatures, it is important to acknowledge that longer-term exposure throughout pregnancy may produce additional or cumulative biological consequences. Prior preclinical and clinical studies have shown that chronic opioid exposure across gestation is associated with impaired placental vascular development, altered trophoblast differentiation, increased inflammatory tone, and higher risk of fetal growth restriction and preterm birth. These placental alterations are believed to contribute to long-term neurodevelopmental vulnerability and metabolic dysregulation in opioid-exposed offspring. While the present study was not designed to model or compare early-, mid-, and late-gestational exposure, the transcriptional signatures identified here, particularly those involving apoptosis, NK-cell-mediated cytotoxicity, cytokine signaling, and extracellular matrix disruption, may represent mechanistic pathways linking opioid exposure to adverse neonatal outcomes observed clinically. Future studies incorporating extended exposure windows and postnatal follow-up will be essential for establishing these developmental trajectories.

## Supplementary Material

Supplementary Material Details

Supplementary Material Details

Supplementary Material Details

Supplementary Material Details

Supplementary Material Details

Supplementary Material Details

Supplementary Material Details
