# Supplementary material for: Maternal opioid use is associated with altered placental bacterial DNA and activation of immune-apoptotic pathways
Source: NeuroImmune Pharm Ther. 2025 Dec 16;4(4):353–62. doi: 10.1515/nipt-2025-0011 (PMC12755123; doi:10.1515/nipt-2025-0011)
Supplement: Supplementary file 5 — Supplementary Material Details [file j_nipt-2025-0011_suppl_005.pptx]

## Slide 1
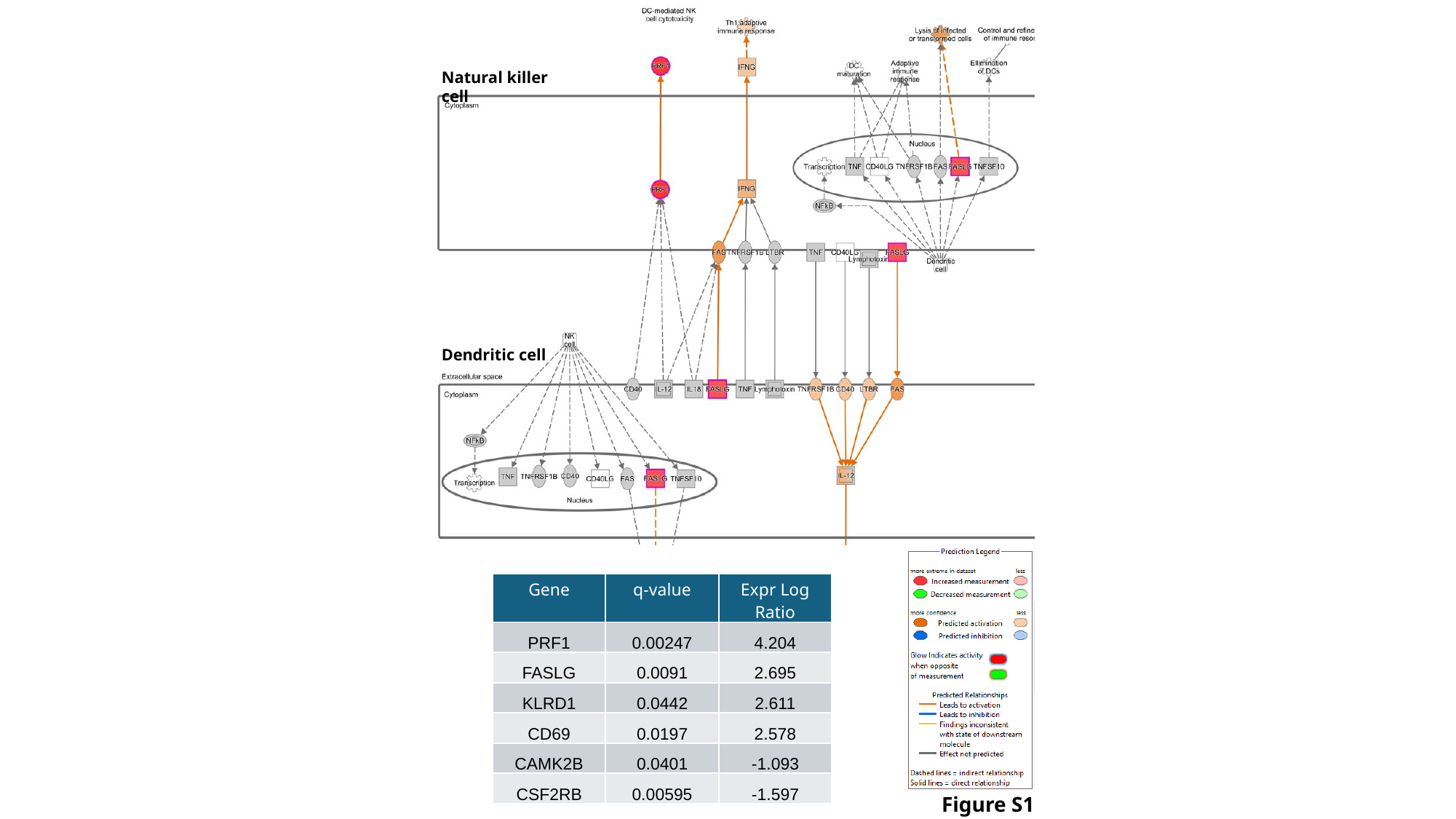

Natural killer cell
Dendritic cell
| Gene | q-value | Expr Log Ratio |
| --- | --- | --- |
| PRF1 | 0.00247 | 4.204 |
| FASLG | 0.0091 | 2.695 |
| KLRD1 | 0.0442 | 2.611 |
| CD69 | 0.0197 | 2.578 |
| CAMK2B | 0.0401 | -1.093 |
| CSF2RB | 0.00595 | -1.597 |
Figure S1

## Slide 2
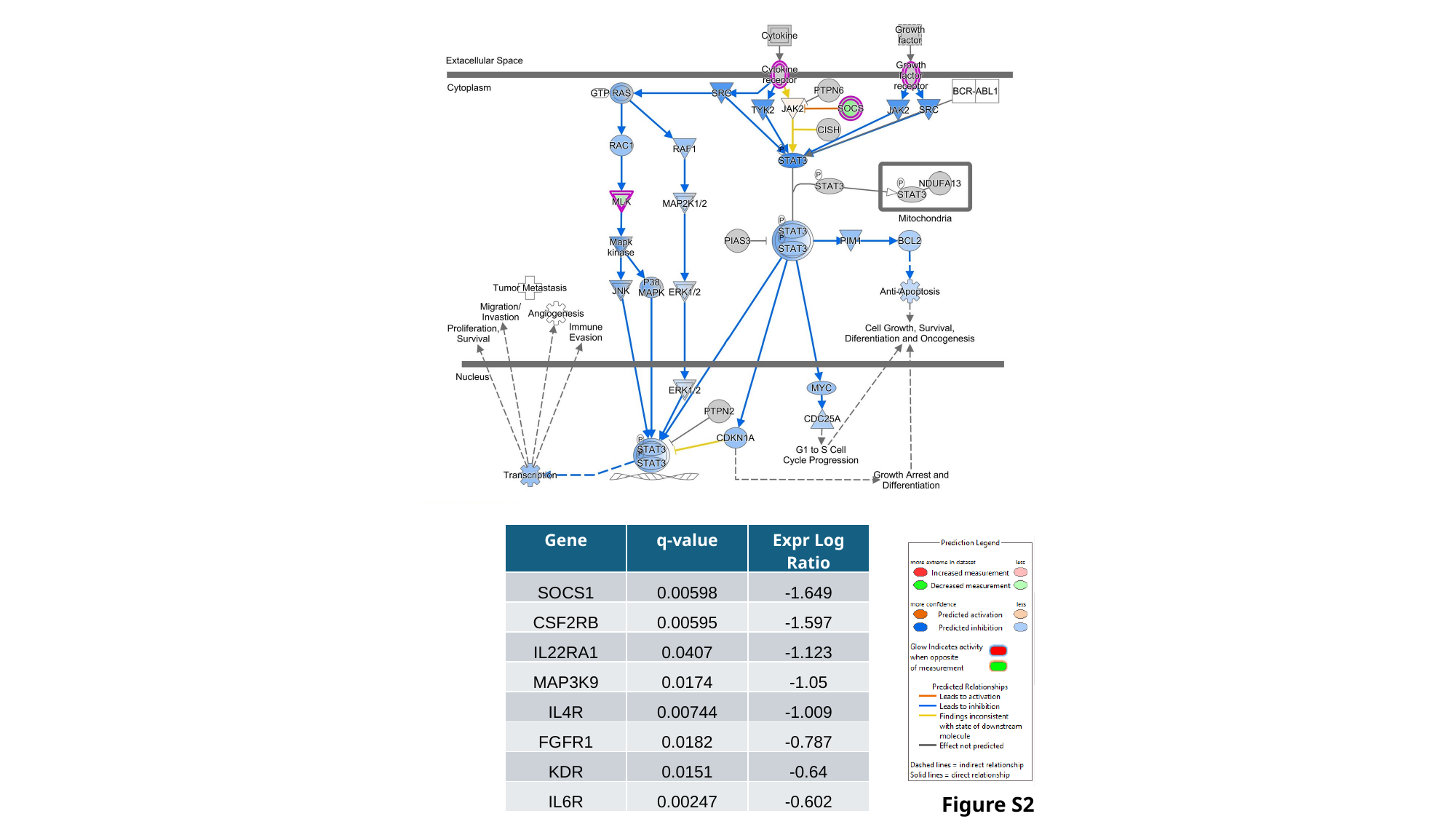

| Gene | q-value | Expr Log Ratio |
| --- | --- | --- |
| SOCS1 | 0.00598 | -1.649 |
| CSF2RB | 0.00595 | -1.597 |
| IL22RA1 | 0.0407 | -1.123 |
| MAP3K9 | 0.0174 | -1.05 |
| IL4R | 0.00744 | -1.009 |
| FGFR1 | 0.0182 | -0.787 |
| KDR | 0.0151 | -0.64 |
| IL6R | 0.00247 | -0.602 |
Figure S2

## Slide 3
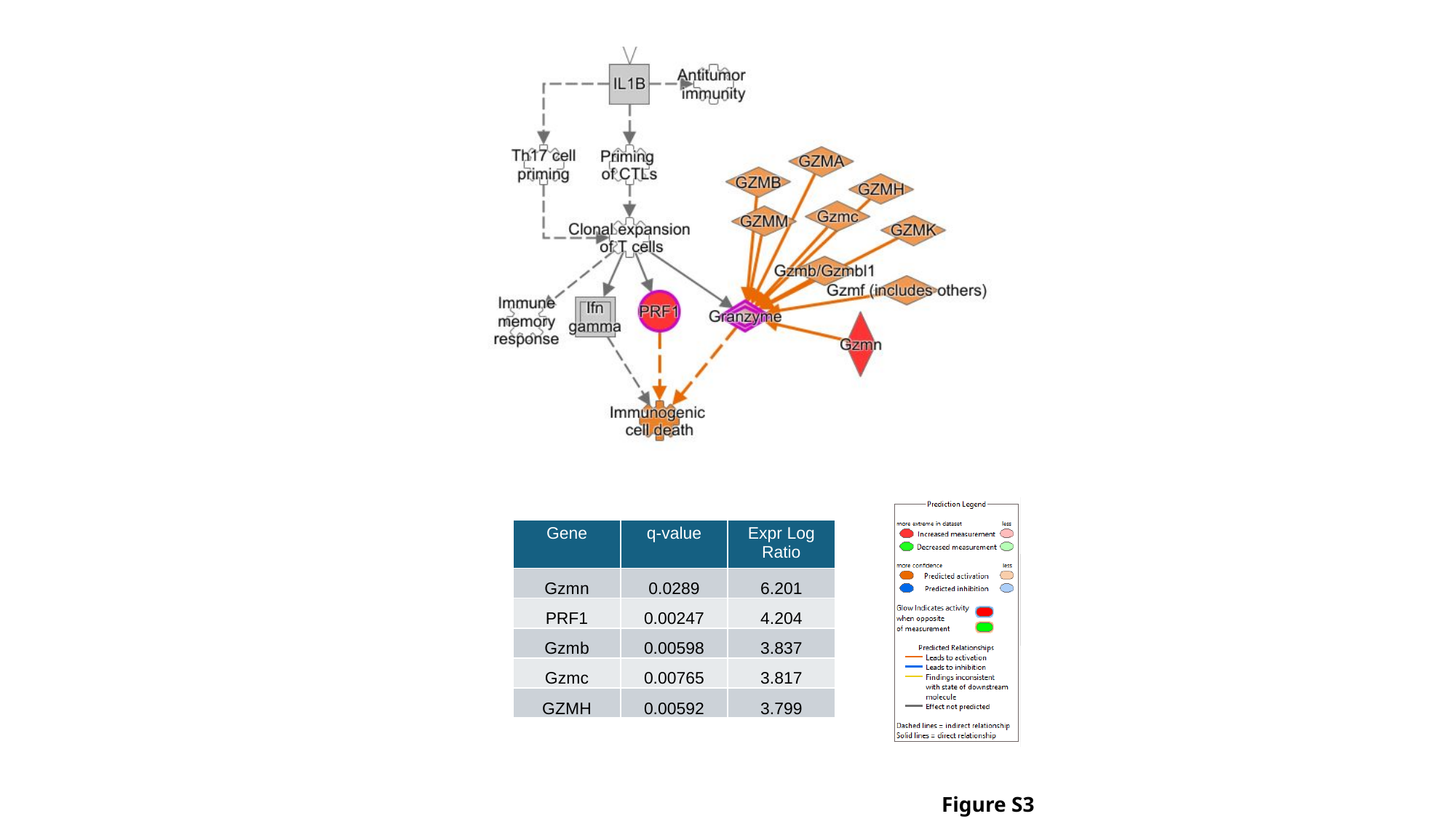

| Gene | q-value | Expr Log Ratio |
| --- | --- | --- |
| Gzmn | 0.0289 | 6.201 |
| PRF1 | 0.00247 | 4.204 |
| Gzmb | 0.00598 | 3.837 |
| Gzmc | 0.00765 | 3.817 |
| GZMH | 0.00592 | 3.799 |
Figure S3

## Slide 4
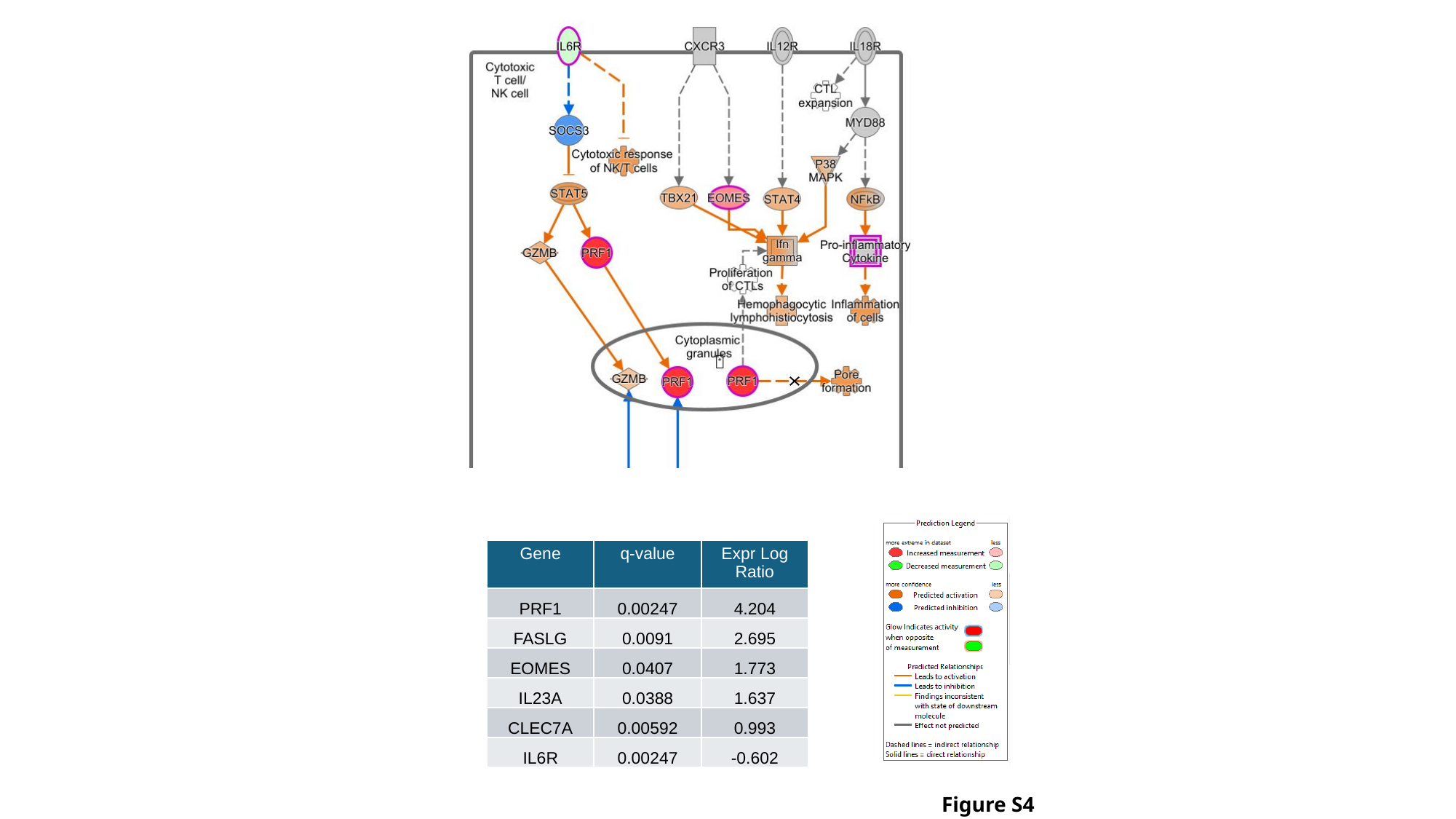

| Gene | q-value | Expr Log Ratio |
| --- | --- | --- |
| PRF1 | 0.00247 | 4.204 |
| FASLG | 0.0091 | 2.695 |
| EOMES | 0.0407 | 1.773 |
| IL23A | 0.0388 | 1.637 |
| CLEC7A | 0.00592 | 0.993 |
| IL6R | 0.00247 | -0.602 |
Figure S4
