# Supplementary material for: Maternal opioid use is associated with altered placental bacterial DNA and activation of immune-apoptotic pathways
Source: NeuroImmune Pharm Ther. 2025 Dec 16;4(4):353–62. doi: 10.1515/nipt-2025-0011 (PMC12755123; doi:10.1515/nipt-2025-0011)
Supplement: Supplementary file 7 — Supplementary Material Details [file j_nipt-2025-0011_suppl_007.docx]

**Figure S1. Detailed view of Crosstalk between Dendritic cells and natural killer cells pathway and significantly expressed gene within the pathway.**

**Figure S2. Detailed view of STAT3 pathway and significantly expressed gene within the pathway.**

**Figure S3. Detailed view of Immunogenic cell death signaling pathway and significantly expressed gene within the pathway.**

**Figure S4. Detailed view of pathogen induced cytokine storm signaling pathway and significantly expressed gene within the pathway.**
